# Effect of different fabrication workflows on the passive fit of screw-retained bar splinting two interforaminal implants: a parallel blinded randomised clinical trial

**DOI:** 10.1186/s12903-024-04157-1

**Published:** 2024-04-02

**Authors:** Bassant Sherif Gamal Eldin, Ingy Amin Talaat, Noha Helmy Hassan Nawar, Ahmed Mostafa Abdelfattah Mohamed

**Affiliations:** Oral and Maxillofacial Prosthodontics Department, Faculty of Dentistry, Ain Shams University, Organization of African Unity Street, Cairo, Egypt

**Keywords:** Dental impression technique, Dental marginal adaptation, Computer-aided design

## Abstract

**Background:**

To clinically compare the effect of the conventional and the digital workflows on the passive fit of a screw retained bar splinting two inter-foraminal implants.

**Methods:**

The current study was designed to be a parallel triple blinded randomised clinical trial. Thirty six completely edentulous patients were selected and simply randomized into two groups; conventional group (CG) and digital group (DG). The participants, investigator and outcome assessor were blinded. In the group (CG), the bar was constructed following a conventional workflow in which an open top splinted impression and a lost wax casting technology were used. However, in group (DG), a digital workflow including a digital impression and a digital bar milling technology was adopted. Passive fit of each bar was then evaluated clinically by applying the screw resistance test using the “flag” technique in the passive and non passive situations. The screw resistance test parameter was also calculated. Unpaired t-test was used for intergroup comparison. P-value < 0.05 was the statistical significance level. The study protocol was reviewed by the Research Ethics Committee in the author’s university (Rec IM051811). Registration of the clinical trial was made on clinical trials.gov ID NCT05770011. An informed consent was obtained from all participants.

**Results:**

Non statistically significant difference was denoted between both groups in all situations. In the passive situation, the mean ± standard deviation values were 1789.8° ± 20.7 and1786.1° ± 30.7 for the groups (CG) and (DG) respectively. In the non passive situation, they were 1572.8° ± 54.2 and 1609.2° ± 96.9. Regarding the screw resistance test parameter, they were 217° ± 55.3 and 176° ± 98.8.

**Conclusion:**

Conventional and digital fabrication workflows had clinically comparable effect on the passive fit of screw retained bar attachments supported by two dental implants.

**Supplementary Information:**

The online version contains supplementary material available at 10.1186/s12903-024-04157-1.

## Background

A statement was produced in 2002 McGill conference that the first choice for the standard of care for the edentulous mandible was an overdenture retained by two implants [1]. Various types of attachments can be used in such overdentures as the bar ones that offer high retentive capacities and help with load distribution [2]. Bars may be either screw or cement retained. Screw retained bars are easier to be retrieved and show better results in the terms of bleeding on probing and low plaque index [3]. However, their passive fit is essential to avoid the biological and mechanical failures as well as the transmission of unfavourable stresses to the bone-implant interface [4].

Several factors contribute to the passive fit of screw retained prosthesis. Such factors include the relative parallelism between the implants, implant number, impression material and technique, in addition to the method of prothesis fabrication [5]. In a study conducted by Narayane et al., they concluded that the more parallel the implants, the more accurate the impression with less deviation of the implant analogs [6]. Michelinakis et al. also reported that less implant deviations were encountered in the short span edentulous sites with a smaller number of implants compared to the long span or the completely edentulous sites having a greater number of implants [7]. Moreover, it was reported that the two-step polyvinyl siloxane impression was significantly less accurate than the one-step putty and light-body combination one [8]. Khan et al. also reported that such combination of impression materials was more accurate than the medium-body polyether impression material. Furthermore, the open top splinted impression technique was stated to be more accurate than the closed top and the non splinted ones [9]. However, a systematic review found no clear evidence that supports the accuracy of one impression technique rather than the others [10]. On the other hand, recent advances in the digital technology within the dental field have innovated the impression techniques. Some studies concluded that the digital impressions were as effective as the conventional ones for the fabrication of the full arch implant supported restorations. However, superior results were attained for the digital ones in case of severe implant angulations [11, 12]. Similarly, another study reported that in cases involving four implants, the digital impressions were found to be more accurate than the conventional ones. Meanwhile, in situations involving a maximum of three implants, the conventional impression was found to be more accurate than the digital one [13].

Screw-retained restorations can be fabricated by the conventional lost wax casting technology. However, they can be also fabricated by a digital technology using an additive method such as laser sintering or a subtractive one such as milling, or combination techniques [14]. The accuracy of the conventional process is limited by the dimensional stability of the various materials used. Furthermore, the wax pattern fabrication is a time-consuming and a labour-intensive step [15]. Therefore, errors during fabrication of the restoration may result and affect its passive fit. To overcome such a problem, digital technology techniques were advocated in the literature [16, 17]. De Franca et al. and Roig et al. concluded that the digitally fabricated screw retained frameworks exhibit better fit when compared to the conventionally fabricated ones [18, 19]. On the other hand, a systematic review reported that the existing scientific clinical evidence does not allow clear conclusions to be drawn about the superiority of the digital technology over the conventional one with respect to the marginal accuracy [20]. Moreover, Abdel-Azim et al., stated that the conventional impression/fabrication workflow resulted in a smaller marginal discrepancy for the single-implant frameworks compared to the digital one that showed better results for the full-arch ones [21, 22].

Various methods are mentioned in the literature for clinical evaluation of the screw retained prosthesis misfit as the alternate finger pressure, radiographic examination and explorer tip. However, such methods are subjective [23, 24]. Rutkunas et al. selected the screw resistance test parameter (SR) in an objective way to evaluate the passive fit of implant-supported restorations clinically. The SR parameter was calculated as the difference of the rotation angles of each screw in the passive and the non passive situations. However, they compared the clinical effect of the impression techniques only with no relevance to the whole workflow [25]. So, the aim of the current study was to clinically compare the passive fit of the screw retained bars fabricated by the conventional and the digital workflows. The null hypothesis tested in this study was that both workflows had no difference in their effect on the passive fit of screw-retained bar attachments.

## Methods

**Fig. 1:**
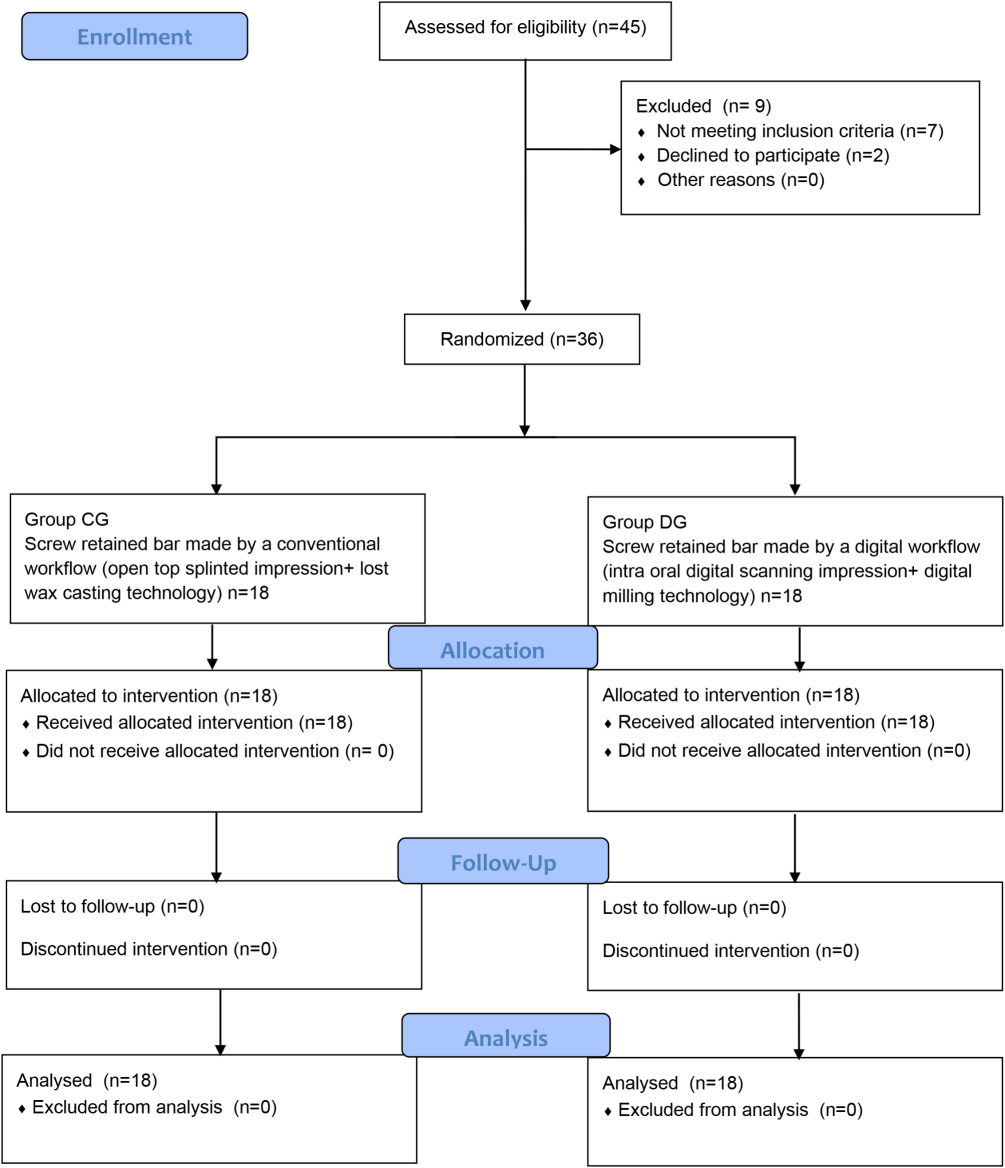
Study flow diagram following the CONSORT guidelines

The current study was designed to be a parallel assigned triple blinded randomized clinical trial following the CONSORT 2010 guidelines. Figure 1 Thirty-six completely edentulous patients were selected to share in the current study. Sample size calculation was based on 95% confidence interval and power 80% with α error 5% (MedCalc® version 12.3.0.0 program, Ostend, Belgium) in the light of a study conducted by Alikhasi et al. [26]. For each patient, two implants were placed in the mandibular right and left canines and received a screw retained bar. Patients were simply randomized using a computer-generated list (Random Alloc, Software informer, Informer Technologies Inc) and allocated into two groups; conventional group (CG) and digital group (DG). For concealment, each patient selected an opaque sealed envelope and the code was revealed to determine the patient’s group. For the group (CG), the bar was fabricated following a conventional workflow that included an open top splinted impression technique coupled with casting technology. However, in the group (DG), a digital workflow including a digital impression and a digital bar milling technology was adopted. Passive fit of each bar was then evaluated intraorally using the screw resistance test [27]. The study protocol was reviewed by the Research Ethics Committee in the author’s university (Rec IM051811). Registration of the clinical trial was made on clinical trials.gov ID NCT05770011 on 15/03/2023. An informed consent was obtained from all participants.

Patients with uncontrolled systematic diseases, heavy smoking habits and conditions that may affect implant osseointegration were excluded. For implant placement, a complete denture was made for each patient. Gutta-percha markers (Meta Biomed, Chungcheong Buk, South Korea) were placed at the mandibular canines to act as radio-opaque markers and a Cone Beam Computed Tomography (CBCT) (i-CAT™ FLX V8, Kavo, California, USA) scan was made for each patient while wearing the denture. A surgical guide was then designed (Blue Sky Plan software, Illinos, USA), printed, stabilized in the patient’s mouth and sequential drilling of the implant sites under copious irrigation then followed [28–30]. The dental implants (Neobiotech Co Ltd, Seoul, Republic of South Korea) were placed in the osteotomy site and three months later the patients were recalled for the second stage surgery and bar fabrication.

For bar fabrication in the group (CG), closed-top primary impressions were made using putty and light addition vinyl polysiloxane impression material in one step (Zermack company, Badia Polisine, Italy). The open top impression posts were then attached to the lab analogs in the study cast and splinted with autopolymerizing acrylic resin (Hard Denture Liner, Promedica GMBH, Germany.). The splint was then split, and the impression posts were resplinted again in the patient’s mouth. The open-top secondary impression was then made using regular body polyvinyl siloxane impression material (Zermack company, Badia Polisine, Italy) [16, 31]. Impressions were made by a single trained operator. Non engaging titanium bases were then screwed to the implant analogs in the master cast (Neobiotech Co Ltd, Seoul, Republic of South Korea) [32]. Readymade wax pattern of the bar (OT bar Rhein83, Bologna, Italy) was then attached to the plastic sleeve of the titanium base and adjusted in position. Lost wax casting technique was then adopted using Co-Cr alloy (BEGO Medical Gmbh, Bremen, Germany). The fitting surface of the bar copings was sandblasted using 110 μm aluminum oxide articles (BEGO sandblaster, BEGO Bremer GMBH, Germany) and primed (Z prime, Bisco, United states of America). Self-cured adhesive resin was then used to cement the bar to the non-engaging Titanium bases (SuperCem, Self adhesive resin cement, Republic of South Korea) [33].

**Fig. 2:**
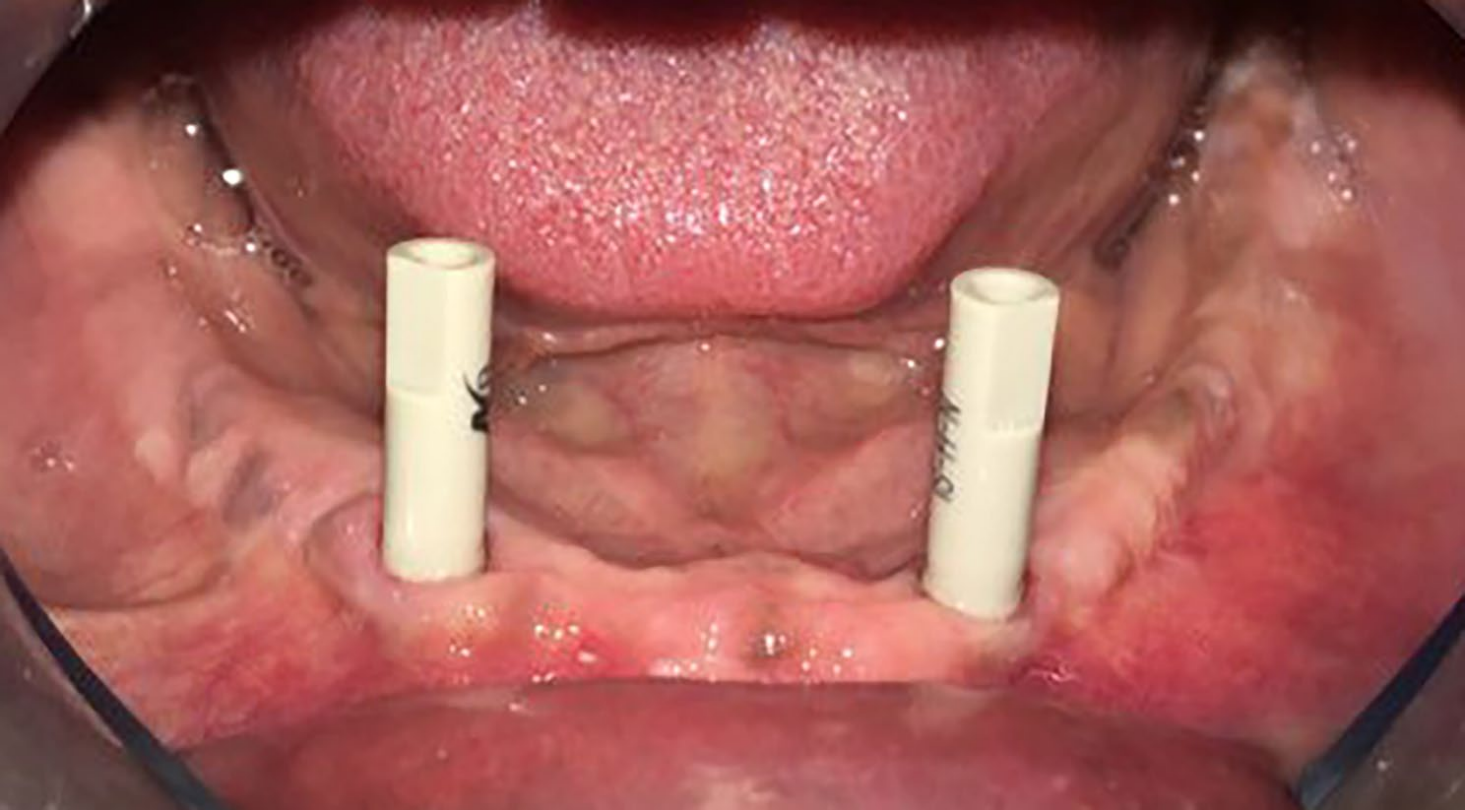
Scan bodies were screwed to the implants ready for intraoral scanning

For bar fabrication in the group (DG), the healing abutments were removed from the implant fixtures in the patient’s mouth. Dryness of the scanning field was essential during the intraoral scanning procedures. Moreover, the patients were instructed to avoid any movements during scanning [34, 35]. Scanning of the lower arch was done using an Omnicam (Dentsply Sirona, Germany) intraoral scanner. Scanning was done by a single calibrated operator who was trained on the intraoral scanning techniques. The scanning process was observed on the computer desktop and if there were any missing data, additional scanning was made. The scan bodies (Neobiotech Co Ltd, Seoul, Republic of South Korea) were then screwed to the implants and scanning was done. Figure 2 [36]. Both scans were previewed on the computer desktop and superimposed together. The Standard tessellation language (STL) file was then exported, downloaded and previewed in Inlab Exocad software (Exocad GMBH, Damastadt, Germany). The implant system was selected from the software library and the restoration type was designed as Rhein 83 OT Bar-A (OT bar Rhein83, Bologna, Italy.). Computer assisted design/computer assisted manufacturing (CAD/CAM) titanium bases (Neobiotech Co Ltd, Seoul, Republic of South Korea) type were selected from the software library. The position of the bar was adjusted in accordance to the titanium bases and the gingiva. The STL file was then exported to the milling machine (Icam v5 imes-icore, Hessen, Germany) and milling of the Co-Cr bar (BEGO Medical Gmbh, Bremen, Germany) was done. Sandblasting and priming of the bar copings and the titanium bases were done in the same steps followed for the group (CG). The bar was then cemented to the non-engaging titanium bases [33, 37].

**Fig. 3:**
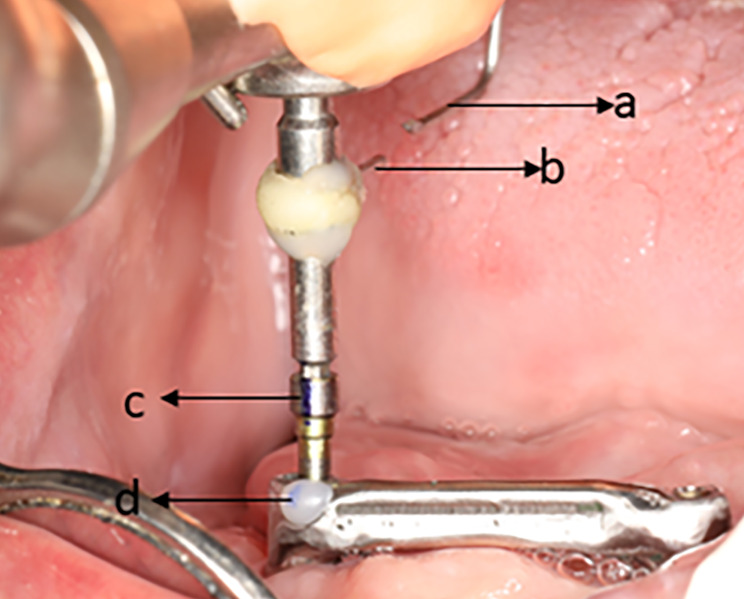
Adjusting the starting position of the pointers for intraoral assessment of the passive fit of the bar using the flag technique. Pointer (**a**) attached to the head of the hand piece denoting the starting position. Pointer (**b**) attached to the shank of the screw driver. Point (**c**) a mark made on the labial surface of the screw. Point (**d**) a mark made on the labial surface of the abutment

Passive fit of each bar was assessed intraorally. Blinding during measurement was done with the help of a third party rather than the outcome assessor. The bars were given to a third party who coded them. The coded bars were given to the trained outcome assessor and the measured values were returned back to the third party to reveal the codes. The screw resistance test was used to evaluate the passive fit of each bar intraorally by using the “flag” technique [27]. The SR parameter was calculated as the difference of the rotation angles of each screw in the passive and the nonpassive situations by the formula: SR = SR passive – SR nonpassive [25]. Tightening of the screws was done using a motor driven screwdriver. One pointer (pointer a) was attached to the head of the hand piece denoting the starting position. A second pointer (pointer b) was attached to the shank of the screwdriver using flowable composite. Both pointers were aligned together at an angle 0° on a protractor. Then, the marks (c and d) were made on the labial surface of the screw and the abutment respectively to be used as a starting point for tightening the screw. Figure 3 First, the right screw was tightened at a torque of 15 Ncm without tightening the left screw to record the SR value in the passive situation [25]. The number of turns rotated by the screw driver’s pointer (pointer b) was counted. The degree of rotation of the last turn was measured by a 360° protractor. For measurement of the last turn degree, the pointer (a) was aligned with the zero degree of the protractor and the angle referred to by the pointer (b) was recorded. The SR passive was calculated by summation of the number of the turns added to the degree of rotation in the last turn. This step was repeated three times and an average reading was recorded. The right screw was then retrieved, and the left screw was tightened at a torque of 15 Ncm. Afterwards, tightening of the right screw was done at a torque of 15 Ncm and the angle of rotation was recorded for the right screw. This value represented the SR value in the nonpassive situation. This step was repeated three times and an average angle was recorded (Video 1). Intra group comparison of the SR passive and non passive situations was done. Inter group comparison of both situations was done as well. Pick up of the bar clips in the reinforced over denture base was made using autopolymerizing acrylic resin [38].

Statistical analysis was performed using the statistical package for social sciences (version 21.0 SPSS Inc; IBM Corporation, Chicago, USA). Shapiro Wilk test revealed normal distribution of the resultant data. Paired t-test was used for the intra group comparison of the SR passive and non passive situations. Unpaired t-test was used for the intergroup comparison for both situations as well as the SR parameter. P-value < 0.05 was considered to be the statistical significance level.

## Results

All participants attended the second stage surgery with no implant loss. The participants in the group (CG) were 9 females and 9 males with average age 67.1 ± 5.6 while in the group (DG), they were11females and 7 males with average age 66.1 ± 5.03. Paired t-test showed statistically significant difference for the intra group comparison of the passive and the non passive situations in both groups (T = 17.1; *P* < 0.001 for group (CG), T = 7.1; *P* < 0.001 for group (DG). On the other hand, Un Paired t-test showed non-statistically significant difference for the inter-group comparison of the passive and non passive situations in both groups. For the passive situation, the T-value was 0.44 while the P-value was 0.33. However, for the non passive situation, the T-value was 1.41 while the P-value was 0.08. Similarly, the Un Paired T test showed non-statistically significant difference for the SR parameter (T = 1.533; *p* = 0.07). The mean and standard deviation values are listed in Table 1.

**Table 1:** Comparison between the groups CG and DG in the passive, non passive situations and the SR parameter using unpaired T-test

	Group CG		Group DG			
	X°	SD	X°	SD	T-test value	P value
Passive	1789.8°	20.7	1786.1°	30.7	0.44	0.33
Non passive	1572.8°	54.2	1609.2°	96.9	1.41	0.08
SR parameter	217°	55.3	176°	98.8	1.53	0.07

X: mean; SD: standard deviation; CG: conventional group; DG: Digital group; SR parameter: screw resistance test parameter (difference of rotation angles in the passive and nonpassive situations: SR = SR passive – SR nonpassive)

## Discussion

The screw retained bar attachment was used in the current study as it allows better stress distribution, provides good retention and can be retrieved easily if biological or technical complications occur [3]. However, the passive fit of such restorations is essential to avoid biological and mechanical complications [4]. So, the current study was performed to clinically compare the passive fit of such attachments fabricated by the conventional and the digital workflows. The null hypothesis that there was no difference between them was accepted as there was no statistically significant difference. Such insignificant difference could be related to the proper selection of the impression materials and techniques, precise steps of bar construction, number of implants and their parallel placement as well as selection of the abutments.

Regarding the impression technique in the group (CG), primary impression was made using the one step putty and light body polyvinyl siloxane impression technique which was reported to be significantly more accurate than the two step one and the medium-body polyether impression material [8, 9]. The adoption of the open top splinted impression coupled with intraoral impression coping resplinting may have prevented individual coping movement during the impression-making procedure and decreased the polymerization shrinkage of the splint material ending up with improved fit of the bar [16, 31]. On the other hand, the inherent limitations in the digital impressions despite being controlled in the group (DG) may have negatively affected the accuracy of such impressions. The humidity of the oral environment, presence of saliva, head movements in addition to accumulation of the registration and angulation errors may have negatively affected the impression’s accuracy as reported in the literature [34, 39]. Moreover, several studies in the literature found no significant differences in the accuracy between both impression techniques. A further randomized clinical trial concluded that the digital impression technique was found to be as effective as the conventional impression technique [12]. Rech. et al. in their study concluded that the conventional impression technique could be more accurate than the digital one for restorations receiving less than three implants [13]. Papaspyridakos et al. also suggested that the digital impressions appear to have comparable three dimensional accuracy with the conventional implant impressions [40]. Furthermore, Rutkunas et al. showed non statistically significant difference between both impression techniques on the passive fit of the implant supported restorations [25]. In another study, it was stated that the accuracy between both impression techniques was found to be comparable in the parallel placed implants; a further reason for the results in the current study as the implants were placed parallel by the help of surgical guides [6, 28]. On the contrary, Roig et al., concluded that the fit of frameworks fabricated by the digital impression technique was better than the conventional one. Such results were owed to the use of a prefabricated auxiliary device in their technique [19].

Regarding the bar construction technology, a systematic review reported that no clear conclusions can be drawn relative to the marginal adaptation about the superiority of the CAD-CAM milling technology as opposed to the conventional casting technique [20]. On the other hand, De Franca et al., stated that the CAD/CAM fabricated frameworks exhibited better fit when compared to the conventionally fabricated ones [18]. However, in such study three implants were placed rather than two as in the current study. Moreover, it was not clear if the implants were aligned parallel in their study as the relative parallelism of the implants could affect the passive fit of the overlying prostheses. Furthermore, Lin et al. concluded that the digital fabrication technology compared to the conventional one resulted in no difference in the accuracy for the parallel placed implants. However, better accuracy was reported for such technology for implants placed at 30 and 45 degrees; a finding that highlights the effect of the parallel placed implants in the current study [41].

Moreover, the cementation of the bars over the non-engaging abutments in both workflows may have accounted for the attained results [32]. Furthermore, the passive fit of the screw retained bar is affected by the number of supporting implants; the less the number of the implants supporting a prothesis, the more passive the fit as concluded by Katsoulis et al. [42]. So, the smaller number of implants used to support the bar in the current study may account for the statistically insignificant difference between both workflows. Furthermore, Abdel-Azim et al., stated that the conventional impression/fabrication workflow resulted in a smaller marginal discrepancy for the single-implant frameworks compared to the digital one that showed better results for full-arch ones [21, 22].

Such less number of the implants used to support the bars in the current study could account for the study limitations. Therefore, further clinical studies evaluating the passive fit of conventionally fabricated screw retained bars supported with more number of implants are recommended. It is also recommended to recruit a larger number of participants in the future studies. Moreover, it has been reported that the use of the nonengaging abutments as in the current study, can produce greater stress in the abutment screws and the implant-abutment interface when forces are applied [43]. Thus, the passive fit in case of using the engaging abutments should be evaluated too. Furthermore, only one type of intraoral scanner has been used in the current study. Therefore, it is also recommended to compare between different types of intraoral scanners with different scanning technologies regarding their effect on the passive fit in the future studies. However, the conventional workflow is cheaper when compared to the digital one. Moreover, intraoral scanners and milling machines are not available in all dental operatories. So, the conventional workflow may provide a cheaper acceptable standard of care for the completely edentulous mandible receiving a two implant retained overdenture, accounting for the clinical relevance of such workflow and matching the 2002 McGill conference statement.

## Conclusion

Conventional and digital fabrication workflows had clinically comparable effect on the passive fit of screw retained bar attachments supported by two dental implants.

### Electronic supplementary material

Below is the link to the electronic supplementary material.

Supplementary Material 1

## Data Availability

The data are available from the corresponding author upon request once the research article becomes published.

## References

[1] Gray D, Patel J (2021). Implant-supported overdentures: part 1. Br Dent J.

[2] Anas El-Wegoud M, Fayyad A, Kaddah A, Nabhan A (2018). Bar versus ball attachments for implant-supported overdentures in complete edentulism: a systematic review. Clin Implant Dent Relat Res.

[3] Hamed MT, Abdullah Mously H, Khalid Alamoudi S, Hossam Hashem AB, Hussein Naguib G (2020). A systematic review of Screw versus Cement-retained fixed Implant supported reconstructions. Clin Cosmet Investig Dent.

[4] Presotto AG, Bhering CL, Mesquita MF, Barão VA (2017). Marginal fit and photoelastic stress analysis of CAD-CAM and overcast 3-unit implant-supported frameworks. J Prosthet Dent.

[5] Kao TY, Hsieh MC, Hsu CP, Liao CC, Chang CL (2023). Accuracy of digital impressions for three-unit and four-unit implant supported fixed dental prostheses using a novel device. J Dent Sci.

[6] Narayane AK, Shamsuddeen S, Kharat S, Rashidi T, Pandav A, Thakur MK (2021). Influence of Implant Angulation and Implant Number on the Accuracy of definitive casts. J Pharm Bioallied Sci.

[7] Michelinakis G, Apostolakis D, Kamposiora P, Papavasiliou G, Özcan M (2021). The direct digital workflow in fixed implant prosthodontics: a narrative review. BMC Oral Health.

[8] Kaur T, Singla S, Kumar L (2023). Comparison of accuracy of hexed and nonhexed pickup impression copings in a multiple variable impression setup for recording multiple straight and angulated implant positions: an in vitro study. J Indian Prosthodont Soc.

[9] Khan SA, Singh S, Neyaz N, Jaiswal MM, Tanwar AS, Singh A (2021). Comparison of Dimensional Accuracy of three different impression materials using three different techniques for Implant impressions: an in Vitro Study. J Contemp Dent Pract.

[10] Kong L, Li Y, Liu Z (2022). Digital versus conventional full-arch impressions in linear and 3D accuracy: a systematic review and meta-analysis of in vivo studies. Clin Oral Investig.

[11] Albayrak B, Sukotjo C, Wee AG, Korkmaz İH, Bayındır F. Three-Dimensional Accuracy of Conventional Versus Digital Complete Arch Implant impressions. J Prosthodont. 2021;2163–70. 10.1111/jopr.13264. 10.1111/jopr.13264 32935894

[12] Cappare P, Sannino G, Minoli M, Montemezzi P, Ferrini F (2019). Conventional versus digital impressions for full Arch Screw-retained Maxillary rehabilitations: a Randomized Clinical Trial. Int J Environ Res Public Health.

[13] Rech-Ortega C, Fernández-Estevan L, Solá-Ruíz MF, Agustín-Panadero R, Labaig-Rueda (2019). C:comparative in vitro study of the accuracy of impression techniques for dental implants: direct technique with an elastomeric impression material versus intraoral scanner. Med Oral Patol Oral Cir Bucal.

[14] Mohajeri M, Khazaei S, Vafaee F, Firouz F, Ghorbani Gholiabad S, Shisheian A (2021). Marginal fit of Temporary restorations fabricated by the Conventional Chairside Method, 3D Printing, and milling. Front Dent.

[15] Ghodsi S, Alikhasi M, Soltani N (2019). Marginal discrepancy of single Implant-supported metal Copings fabricated by various CAD/CAM and conventional techniques using different materials. Eur J Dent.

[16] Hashemi AM, Hashemi HM, Siadat H, Shamshiri A, Afrashtehfar KI, Alikhasi M (2022). Fully Digital versus Conventional workflows for fabricating posterior three-unit Implant-supported reconstructions: a prospective crossover clinical trial. Int J Environ Res Public Health.

[17] Alikhasi M, Alsharbaty MHM, Moharrami M (2017). Digital implant impression technique accuracy: a systematic review. Implant Dent.

[18] De França DG, Morais MH, das Neves FD, Carreiro AF, Barbosa GA (2017). Precision Fit of Screw-Retained Implant-supported fixed Dental Prostheses fabricated by CAD/CAM, Copy-Milling, and conventional methods. Int J Oral Maxillofac Implants.

[19] Roig E, Roig M, Garza LC, Costa S, Maia P, Espona J (2022). Fit of complete-arch implant-supported prostheses produced from an intraoral scan by using an auxiliary device and from an elastomeric impression: a pilot clinical trial. J Prosthet Dent.

[20] Papadiochou S, Pissiotis AL (2018). Marginal adaptation and CAD-CAM technology: a systematic review of restorative material and fabrication techniques. J Prosthet Dent.

[21] Hayama H, Fueki K, Wadachi J, Wakabayashi N (2018). Trueness and precision of digital impressions obtained using an intraoral scanner with different head size in the partially edentulous mandible. J Prosthodont Res.

[22] Abdel-Azim T, Zandinejad A, Elathamna E, Lin W, Morton D (2014). The influence of digital fabrication options on the accuracy of dental implant-based single units and complete-arch frameworks. Int J Oral Maxillofac Implants.

[23] Katsoulis J, Takeichi T, Sol Gaviria A, Peter L, Katsoulis K (2017). Misfit of implant prostheses and its impact on clinical outcomes. Definition, assessment and a systematic review of the literature. Eur J Oral Implantol.

[24] Abdelrehim A, Etajuri EA, Sulaiman E, Sofian H, Salleh NM (2022). Magnitude of misfit threshold in implant-supported restorations: a systematic review. J Prosthet Dent.

[25] Rutkunas V, Larsson C, von Vult P, Mangano F, Gedrimiene A (2020). Clinical and laboratory passive fit assessment of implant-supported zirconia restorations fabricated using conventional and digital workflow. Clin Implant Dent Relat Res.

[26] Alikhasi M, Siadat H, Nasirpour A, Hasanzade M. Three-Dimensional Accuracy of Digital Impression versus Conventional Method: Effect of Implant Angulation and connection type. Int J Dent. 2018;3761750. 10.1155/2018/3761750. 10.1155/2018/3761750 PMC6008832 29971107

[27] Kan JY, Rungcharassaeng K, Bohsali K, Goodacre CJ, Lang BR (1999). Clinical methods for evaluating implant framework fit. J Prosthet Dent.

[28] Pessoa R, Siqueira R, Li J, Saleh I, Meneghetti P, Bezerra F, Wang HL, Mendonça G (2022). The impact of Surgical Guide fixation and Implant Location on Accuracy of Static Computer-assisted Implant surgery. J Prosthodont.

[29] Wang M, Rausch-Fan X, Zhan Y, Shen H, Liu F (2022). Comparison of Implant Placement Accuracy in Healed and fresh extraction sockets between static and dynamic computer-assisted Implant surgery Navigation systems: a model-based evaluation. Mater (Basel).

[30] Fraguas de San José L, Ruggeri FM, Rucco R, Zubizarreta-Macho Á, Alonso Pérez-Barquero J, Riad Deglow E (2020). Hernández Montero S:influence of drilling technique on the Radiographic, Thermographic, and Geomorphometric effects of Dental Implant drills and Osteotomy Site preparations. J Clin Med.

[31] Lyu M, Di P, Lin Y, Jiang X (2022). Accuracy of impressions for multiple implants: a comparative study of digital and conventional techniques. J Prosthet Dent.

[32] Schoenbaum TR, Stevenson RG, Balinghasay E (2018). The hemi-engaging fixed dental implant prosthesis: a technique for improved stability and handling. J Prosthet Dent.

[33] Fouquet V, Dantagnan CA, Abdel-Gawad S, Dursun E, Attal JP, François P (2023). In vitro shear bond strength over zirconia and titanium alloy and degree of conversion of extraoral compared to intraoral self-adhesive resin cements. BDJ Open.

[34] Kachhara S, Nallaswamy D, Ganapathy DM, Sivaswamy V, Rajaraman V (2020). Assessment of intraoral scanning technology for multiple implant impressions - a systematic review and meta-analysis. J Indian Prosthodont Soc.

[35] Schmalzl J, Róth I, Borbély J, Hermann P, Vecsei B (2023). The impact of software updates on accuracy of intraoral scanners. BMC Oral Health.

[36] Latham J, Ludlow M, Mennito A, Kelly A, Evans Z, Renne W (2020). Effect of scan pattern on complete-arch scans with 4 digital scanners. J Prosthet Dent.

[37] Aljohani MS, Bukhari HA, Alshehri M, Alamoudi A (2022). Accuracy of the different materials used to fabricate a Verification Jig of Implant-supported fixed complete Dental prostheses: an in Vitro Study. Cureus.

[38] Nassar HI, Abdelaziz MS (2022). Retention of bar clip attachment for mandibular implant overdenture. BMC Oral Health.

[39] Wulfman C, Naveau A, Rignon-Bret C (2020). Digital scanning for complete-arch implant-supported restorations: a systematic review. J Prosthet Dent.

[40] Papaspyridakos P, Vazouras K, Chen YW, Kotina E, Natto Z, Kang K, Chochlidakis K (2020). Digital vs Conventional Implant impressions: a systematic review and Meta-analysis. J Prosthodont.

[41] Lin WS, Harris BT, Elathamna EN, Abdel-Azim T, Morton D (2015). Effect of implant divergence on the accuracy of definitive casts created from traditional and digital implant-level impressions: an in vitro comparative study. Int J Oral Maxillofacial Implants.

[42] Katsoulis J, Müller P, Mericske-Stern R, Blatz MB (2015). CAD/CAM fabrication accuracy of long- vs. short-span implant-supported FDPs. Clin Oral Implants Res.

[43] Savignano R, Soltanzadeh P, Suprono MS (2021). Computational biomechanical analysis of engaging and nonengaging abutments for Implant Screw-retained fixed Dental Prostheses. J Prosthodont.

